# Impact of Percutaneous Coronary Intervention and Implantation of Intra-Aortic Balloon Pump on the Outcome of an Acute Total Obstruction of the Left Main Coronary Artery

**DOI:** 10.1155/2021/5522501

**Published:** 2021-07-29

**Authors:** Hamza Hamayel, Yahya Ismail, Sajed Majadla, Yousef Hamshari, Yunis Daralammouri

**Affiliations:** ^1^Department of Internal Medicine, An-Najah National University Hospital, Nablus, State of Palestine; ^2^Department of Cardiology, An-Najah National University Hospital, Nablus, State of Palestine; ^3^Department of Medicine, Faculty of Medicine and Health Sciences, An-Najah National University, Nablus, State of Palestine

## Abstract

**Background:**

Acute total occlusion of the left main coronary artery (LMCA) is a fatal event; most patients die before reaching hospitals. Few of them reach the hospital alive. Revascularization of the LMCA can be achieved by surgical intervention or percutaneous coronary intervention with unknown optimal modality. However, mortality of those patients is very high even with either; few cases reported successful management of acute total occlusion of the LMCA including our patient. *Case Presentation*. A 56-year-old male patient who is a smoker presented with typical chest pain worsened 2 hours prior to admission. He was hemodynamically stable, but he had respiratory failure due to pulmonary edema. An electrocardiogram showed anterior ST-elevation myocardial infarction. He was given loading doses of dual antiplatelet agents, in addition to respiratory support, then transferred immediately to the cardiac catheterization laboratory. Urgent cardiac catheterization showed total occlusion of the LMCA. Recanalization was done successfully, and a stent was inserted in the LMCA and left anterior descending artery. The patient developed cardiogenic shock during the procedure. An intra-aortic balloon pump (IABP) was applied which improved his hemodynamic status and enhanced his coronary flow. He is clinically improved, there was resolution of ST elevation, and cardiogenic shock gradually resolved. IABP was removed, and the patient was discharged in good general condition.

**Conclusions:**

Survival after acute total occlusion of the LMCA is very rare. The good outcome in this patient is attributed to early recognition and timely successful intervention, with good respiratory and hemodynamic support. The surgical and anaesthesia team should be on stand-by until complete revascularization and stabilization of the patient are achieved.

## 1. Background

Acute total occlusion of the left main coronary artery (LMCA) is an emergency condition and a catastrophic event. It is a very rare presentation of acute coronary syndrome [1]. Sudden death due to cardiogenic shock or arrhythmia is usually the outcome which occurred before any intervention can be done [2, 3]. The remaining patients mostly presented with cardiogenic shock and critical condition with a very high mortality rate even with revascularization [4].

Literature case reports revealed successful management of acute total occlusion of the LMCA with coronary artery bypass graft (CABG) and percutaneous coronary intervention (PCI). However, optimal modality is unknown yet [5]. There is limited data about the presentation with ST-elevation myocardial infarction (STEMI) and cardiogenic shock.

Our report describes a 56-year-old man with acute total occlusion of the LMCA presenting as ST-elevation myocardial infarction (STEMI) that developed cardiogenic shock during the procedure and treated with emergency primary PCI and simultaneous implantation of IABP.

## 2. Case Presentation

**Figure 1:**
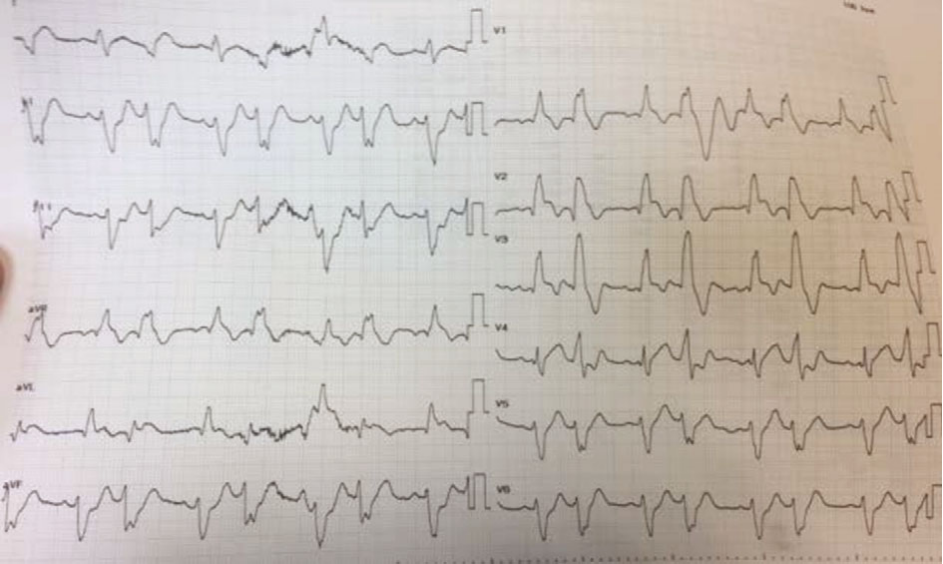
Electrocardiogram at presentation: sinus rhythm, STEMI in anterior chest leads, left axis deviation, PVCs in bigeminy pattern.

In December 2018, a 56-year-old male patient who is a heavy smoker without other comorbidities was referred to our hospital as a case of acute coronary syndrome for cardiac catheterization. The history of the patient revealed that he had typical retrosternal chest pain for 8 hours increased in intensity 2 hours prior to hospital admission. He received his first medical assessment 40 minutes before presentation to our hospital where he was given 300 mg chewable aspirin, 300 mg clopidogrel, and 5000 international unit (IU) of heparin for a diagnosis of STEMI by electrocardiogram (ECG). Upon arrival to our hospital, he was conscious but in severe respiratory distress in terms of tachypnea and hypoxemia, in addition to anginal chest pain. Blood pressure was 135/90 mmHg, and heart rate was 50 beats/min with regular pulse, SpO_2_ 75% on room air. Physical examination showed elevated jugular venous pressure (JVP), bilateral chest crepitation up to mid-chest-zones, and unremarkable cardiac auscultation findings. Pulmonary edema was diagnosed, and oxygen was started by nonrebreather mask (NRM) along with intravenous (IV) furosemide and nitroglycerine infusion, to relieve chest pain and improve respiratory condition. ECG showed left axis deviation, and STEMI in the anterior chest leads with premature ventricular complexes (PVCs) in bigeminy pattern (Figure 1). The patient was transferred to the cardiac catheterization laboratory for primary PCI.

**Figure 2:**
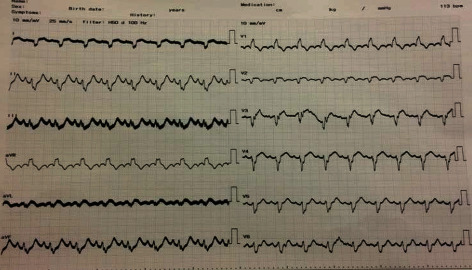
Electrocardiogram after primary PCI: it showed resolution of STEMI.

**Figure 3:**
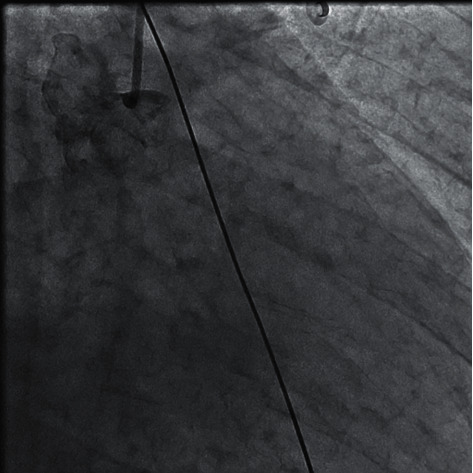
Cardiac catheterization showed total occlusion of LMCA.

**Figure 4:**
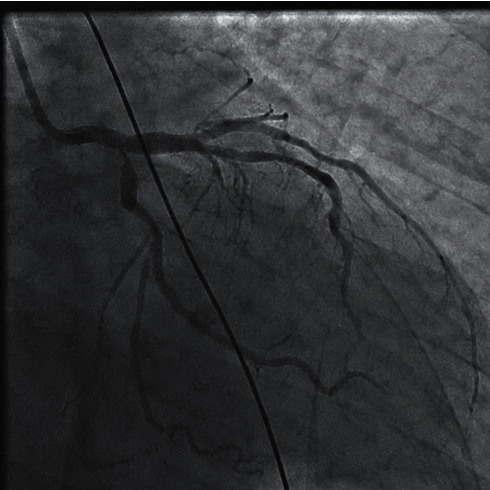
Left coronary artery after stenting LMCA with restoration of blood flow.

The door to balloon time was eighty minutes. During preparation for cardiac catheterization, his symptoms from pulmonary edema did not improve. So, we put the patient on noninvasive ventilation which resulted in significant improvement of his respiratory symptoms. We proceeded with cardiac catheterization using the left Judkins guide catheter through femoral access; the 1st injection of contrast material revealed total occlusion of the left main coronary artery (LMCA) with thrombolysis in myocardial infarction (TIMI) flow “0” (Figure 2). Meanwhile, the cardiac surgery and anaesthesia team was called and remained on standby in case CABG surgery become necessary. Successful Recanalization of the Left Coronary Circulation LCS using 0.014 × 180 cm floppy guide wire, subsequently predilation with 2.00 × 20 mm balloon was done followed by deployment of 4.00 × 28 mm everolimus drug-eluting stent (Figure 3). During intervention, he developed hypotension which required starting norepinephrine infusion, dose titrated up to 0.15 mcg/kg/min to keep mean arterial BP over 65 mmHg. Also, he developed two attacks of ventricular tachycardia with hypotension terminated with defibrillation. He was started on amiodarone infusion. Lidocaine infusion was added after the second attack of ventricular tachycardia. As soon as the procedure on the left coronary artery was completed, the right coronary artery was examined which showed a dominant artery with significant proximal stenosis, and moderate diffuse stenosis from mid to distal segment (Figure 4). On the following 24 hours, the patient maintained on heparin infusion at a rate of 1000 unit/hour titrated on the basis of activated thromboplastin time (aPTT) to achieve twice the normal value, and tirofiban with initial of bolus of 25 microgram/kg over 3 minutes followed by continuous infusion at a rate of 0.15 mcg/kg/min.

**Figure 5:**
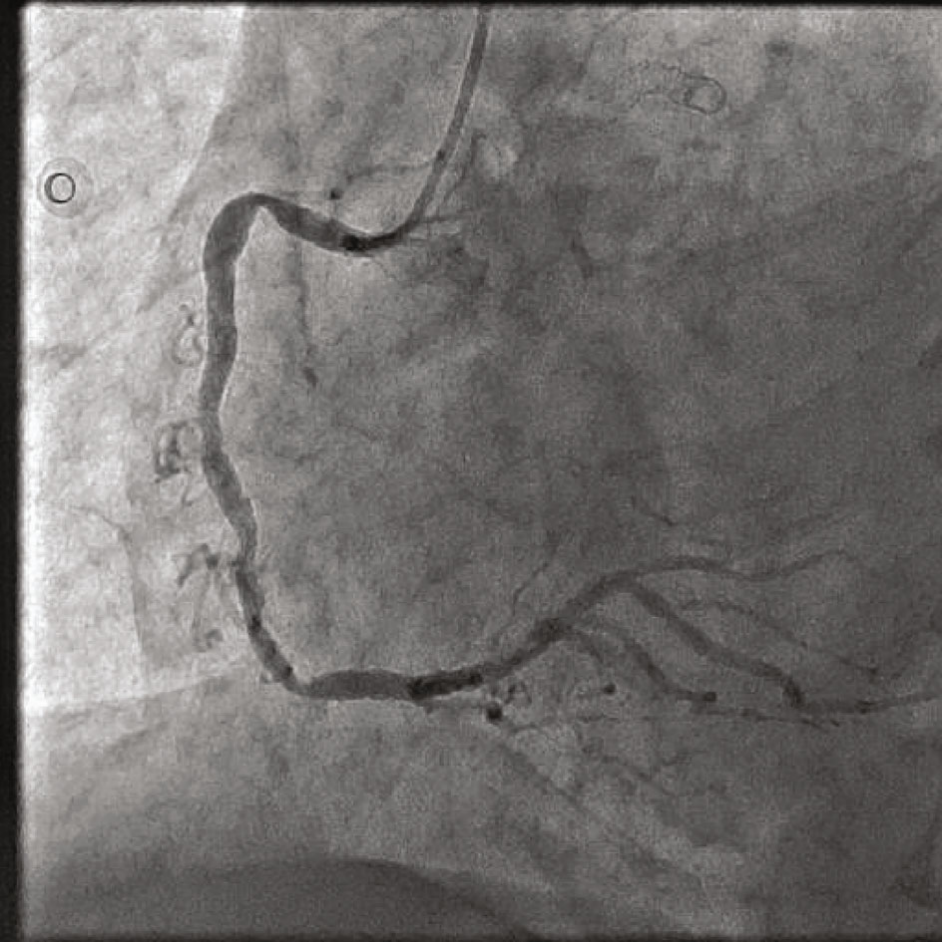
Cardiac catheterization for right coronary artery, dominant artery and showed proximal significant stenosis, and moderate diffuse stenosis from mid to distal segment.

At the end of the procedure, the patient remained hypotensive with slow coronary flow. An intra-aortic balloon pump (IABP) was positioned under fluoroscopy via femoral access, and 1 : 1 augmentation was started. On admission, troponin was normal. Six hours after the procedure, troponin was 1028 ng/ml, reference range (0.1-0.4). ECG showed resolving of ST-elevation and PVCs with a bigeminy pattern (Figure 5). After the PCI, blood pressure started to improve. Two days after intervention, he was weaned from IABP, and norepinephrine infusion was tapered down gradually till we stopped it on day four; troponin continued to decrease gradually till it became negative, and Transthoracic Echocardiography (TTE) after removal of IABP showed ejection fraction (EF) 40%. The patient was discharged after 8 days of hospitalization in good general condition with stable vital signs on the following medications: aspirin 100 mg once daily, clopidogrel 75 mg twice daily for the first 2 weeks then once daily, atorvastatin 80 mg once daily, furosemide 40 mg once daily, bisoprolol 2.5 mg once daily, and enalapril 5 mg once daily.

## 3. Discussion

Total occlusion of the LMCA is a rare and fatal event. It is reported in less than 0.6% of acute coronary syndrome who were admitted for primary PCI. Most of them die before arriving to the hospital. While mortality for those patients is very high even with appropriate management, only half of the patients survived and discharged alive from the hospital [1].

As the LMCA supplies most of the left ventricular mass, acute occlusion will lead to catastrophic massive myocardial infarction and arrhythmia. This explain differences in the outcome and mortality when it is compared with occlusion of distal coronary bed especially in the absence of collateral circulation [1].

It is still unknown what is the best management of such cases, either to proceed with PCI or CABG [5]. Successful revascularization by angioplasty and stenting of the LMCA was reported with a good final result [6–9]. Emergency CABG is also effective in restoring blood flow, but it is time consuming and may carry risk of irreversible damage by ischemia [9]. There are reported cases where PCI failed and the patient underwent emergency CABG to restore blood flow [10]. However, this could be different right now with the improvement of intervention technique and stent technology. In our case, ECG for the patient was confusing and its changes did not give any hint for total occlusion of the LMCA. So, when the first injection of contrast material revealed total occlusion of the left main coronary artery, it was obvious that PCI is the appropriate approach for time interest. Meanwhile, the cardiac surgery and anaesthesia team was called to be on standby for any need for urgent CABG surgery. Fortunately, our hospital is a tertiary hospital, well-staffed, and with equipment for primary PCI. Opening of the occlusion and restoration of blood flow was successfully done within 80 minutes.

Periprocedure cardiogenic shock, no flow after intervention, and late presentation were reported as a poor prognostic factor and carry a high mortality rate [1]. Our case survived because of early recognition, timely preparation of cardiac catheterization laboratory, and successful revascularization. This was a result of good and proper communication between emergency room doctors, cardiologist on call, and cardiac catheterization laboratory team. Moreover, early supportive management of respiratory failure with noninvasive ventilation and early treatment of pulmonary edema in addition to hemodynamic support using norepinephrine and IABP kept the patient stable until reperfusion was achieved.

Hypotension during the procedure as a result of reperfusion injury was suspected before revascularization. For which, femoral access was selected instead of radial access, and vasopressor and IAPB were prepared before reperfusion. Hemodynamic support with norepinephrine was the initial vasopressor according to guidelines [11]. It was started during intervention once his blood pressure started to drop before any hypotension episode; then, it was titrated according to mean arterial pressure reading keeping it more than 65 mmHg. After reperfusion was done and due to slow flow (TIMI 1 flow), IABP was applied as stabilizing measure and to enhance coronary perfusion. There is conflicting result about the benefit of IABP in cardiogenic shock [12]. In the IABP-SHOCK II trial, there was no difference in the outcome between patients that underwent IABP insertion and those who did not [13]. However, this cannot be generalized to LMCA disease which constitutes 9% of cases in that trial. Moreover, it is usually inserted when other devices like the left ventricular assistance device (LVAD) are not available.

In conclusion, this report describes a 56-year-old male patient who survived acute LMCA occlusion treated with primary PCI. Early recognition and timely intervention with appropriate management resulted in good outcome. The patient survived until discharge from the hospital. At 2-year follow-up, he remained in good general condition.

## Data Availability

All data supporting the study is presented in the manuscript or available upon request from the corresponding author of this manuscript.

## Abbreviations

LMCA: Left main coronary artery
ECG: Electrocardiogram
STEMI: ST-elevation myocardial infarction
CABG: Coronary artery bypass graft
PCI: Percutaneous coronary intervention
IABP: An intra-aortic balloon pumps (IABP)
PVC: Premature ventricular complex.

## Supplementary Materials

Supplementary Materials Coronary artery angiography (cardiac catheterization) for the acute total occlusion of the left main coronary artery. It involves percutaneous coronary intervention and stenting of LMCA.
